# Sensitivity analysis of human error in the steel industry: exploring the effects of psychosocial and mental health risk factors and burnout using Bayesian networks

**DOI:** 10.3389/fpubh.2024.1437112

**Published:** 2024-10-08

**Authors:** Saeid Yazdanirad, Amir Hossein Khoshakhlagh, Saleh Al Sulaie, Rosanna Cousins, Mohammad Dehghani, Reza Khodakhah, Saeid Shabanitabar

**Affiliations:** ^1^Department of Occupational Health, School of Health, Shahrekord University of Medical Sciences, Shahrekord, Iran; ^2^Department of Occupational Health, School of Health, Kashan University of Medical Sciences, Kashan, Iran; ^3^Department of Mechanical and Industrial Engineering, College of Engineering and Computers in Al-Qunfudah, Umm Al-Qura University, Makkah, Saudi Arabia; ^4^Department of Psychology, Faculty of Human and Digital Sciences, Liverpool Hope University, Liverpool, United Kingdom; ^5^Student Research Committee, Kashan University of Medical Sciences, Kashan, Iran; ^6^Independent Researcher, Kashan, Iran

**Keywords:** accident prevention, depersonalization, emotional exhaustion, employee stress, steel industry

## Abstract

**Introduction:**

Human error and the high rates of fatalities and other occupational accidents in the steel industry are of significant global relevance. The aim of this study was to investigate the effect of psychosocial, mental health, and burnout risk factors on human error probabilities in an industrial environment using Bayesian networks.

**Methods:**

This cross-sectional study was conducted in 2023. The participants were 252 employees of a steel company. Error probabilities related to the tasks of participants were estimated using the Human Error Assessment and Reduction Technique (HEART). Other data was collected using a survey that consisted of demographic information, the Maslach Burnout Inventory, Depression Anxiety Stress Scales, and a short version of the Copenhagen Psychosocial Questionnaire. A theoretical model was drawn in GeNIe academic software (version 2.3).

**Results:**

The results showed that all the studied variables were able to significantly affect the distribution of human error probabilities. Considering a distribution of 100% for the high state of these variables, the results showed that the greatest increases in error probability were related to two burnout dimensions: emotional exhaustion (29%) and depersonalization (28%). All the variables, with a probability of 100%, increased the probability of high human error probabilities by 46%.

**Conclusion:**

The most important variables in terms of their effect on human error probabilities were burnout dimensions, and these variables also had a mediation effect on the psychosocial and mental health variables. Therefore, preventive measures to control human error should first focus on managing the risks of burnout in workers. This, in turn, can also reduce the effect of psychosocial risk factors and mental health problems on human error in the workplace.

## Introduction

Industrial accidents remain an important problem in many of today’s societies (1). According to International Labor Organization statistics, 321,000 people die every year due to occupational accidents and there are 310 million accidents with various non-fatal injuries around the world (2). These accidents occur particularly in dangerous industries, such as steel companies. The results of a study performed by Berhan showed that the prevalence of occupational accidents in iron, steel, and metal manufacturing industries is relatively high (3). Fuentes-Bargues et al. concluded that the metal sector has a high percentage of serious and fatal accidents (4). Nazari and Dashti observed that occupational accidents can affect the quality of working life in Iranian workers of a steel company (5).

It is known that the underlying reason for most occupational accidents—about 90%—comes from unsafe actions and behaviors (6), and among the types of unsafe behaviors, human errors are one of the main causes of such accidents (7). Understanding the variables that contribute to human errors in work environments is necessary for any effective plan to reduce occupational accidents (8). Ultimately, anything that can disrupt human mental performance and concentration and cause decreased human cognitive ability can play an important role in creating human errors (9).

There are studies that have investigated the effect of psychosocial risk factors on occupational accidents caused by human error and confirmed that they are an important accident agent (10, 11). Psychosocial risk factors are defined as employees’ perceptual experiences of the quality of organizational environments and working conditions (12). They are psychological in nature and can lead to stress, alongside mental and behavioral problems (13). Similarly, mental health problems can be agents of human errors through changes in thinking, mood, emotion, or behavior and disruption in life functioning that define these conditions (14). Among mental health issues, depression, anxiety, and stress play a large role (15), and conversely, it has been shown that management of stress and anxiety can effectively decrease the probability of human error and improve safety in safety-critical workplaces (16). There is also evidence from a prospective study that compared depressed and non-depressed pediatric residents found that the former made significantly more medical errors (17), highlighting the importance of managing employees’ mental health. Barkhordari et al. investigated individual and social factors affecting occupational accidents in a steel company and concluded that psychosocial factors can affect occupational accidents through occupational stress (18). The results of a study performed by Rabiei et al. showed that psychosocial factors and job stress could influence unsafe behaviours in the workers of a steel company (19). Moreover, Barkhordari et al. observed that individuals working in the steel industry became more vulnerable to the accidents and unsafe behaviors because of occupational stress, effort-reward imbalance, and work family conflict (20).

Burnout is another variable that can influence human errors. Burnout is an occupational phenomenon caused by chronic job stress. It is a syndrome associated with feelings of complete lack of energy; feeling useless, powerless, and empty, detached from one’s job, which ultimately leads to reduced efficacy in the work being done (21). There is evidence of a significant relationship between burnout and human errors in healthcare (22), and task performance (23, 24).

In addition to the effect of psychosocial, mental health, and burnout variables on human error, there are internal relationships between these variables. For example, Lindblom et al. conducted an investigation of relationships between psychosocial risk factors and burnout among working populations and concluded that occupational psychosocial factors are important in the relationship to burnout regardless of job (25). The literature also includes reports of significant relationships between psychosocial work factors and major depressive disorder and generalized anxiety disorder (26), and between psychosocial risk factors and work-related stress (27). Also, a systematic review and meta-analysis study conducted by Koutsimani et al. indicated that there are significant associations between depression and anxiety with burnout (28). Therefore, it may be that there are interactions between these factors that further impress on, or mediate, human errors. Identification of these interactions and their importance will also be very useful for planning preventive measures.

To date, there has been no previous research that has explored the interrelations among these variables within a comprehensive model. Also, previous studies have not quantified the size of their effects through different pathways. Bayesian networks, which use inherently probabilistic and graphical models, are an effective analytical tool for this purpose (29). Bayesian networks employ probabilistic reasoning to manage uncertainties, and rules of probability for both learning and making inferences (30). In these networks, random variables and their conditional interdependencies are represented visually. The strength of Bayesian networks lies in their ability to assess an event by calculating the likelihood of its occurrence due to various possible influences (31). Bayesian networks have been utilized across a range of fields, from safety and health to decision support systems, although most previous studies have been performed in medical environments. Safety at work has a broader importance than healthcare, therefore this study aimed to investigate the effect of psychosocial, mental health, and burnout risk factors on human error probabilities in an industrial environment using Bayesian networks. In particular, there is a paucity of investigations in the steel sector, which recorded 85 fatalities, and 18,448 injuries across the globe in 2022 (32). Thus, in view of this need to extend the literature, and provide a comprehensive examination of the contribution of psychosocial risk factors, stress, and burnout to human error that is relevant beyond medical error, this study was conducted in a large steel plant.

## Methods

This cross-sectional study was conducted in 2023, in a steel company located in the center of Iran. The research ethics committee of Kashan University of Medical Sciences (IR.KAUMS.MEDNT.REC.1402.204).

### Participants

Participants were recruited from a large steel factory located in Kashan, Iran, utilizing a random sampling technique. The parameters for inclusion mandated that participants be between the ages of 18 and 60, possess at least 1 year of professional experience, and have no history of mental health conditions. Exclusion criteria were a lack of willingness to provide informed consent to join the study and submission of an incomplete survey.

During the study recruitment phase, a comprehensive roster encompassing 750 workers from the factory was compiled. From this list, 300 employees in a range of roles in steel fabrication were initially approached (according to the inclusion criteria), provided with information regarding the objectives and methodology of the study, and invited to join the study. Out of these invitees, 252 individuals provided informed consent and submitted a fully filled-out survey, resulting in a participation rate of 84%.

### Data collection

First, relevant parts of the steel factory were visited by the researchers, and the tasks related to the participants’ work were determined. Then, error probabilities related to these tasks were estimated by the human error assessment and reduction technique (HEART). In order to collect the remaining required data, consenting participants were asked to complete a paper-based survey during a rest break. Assistance from the researchers was available for any queries or help needed in filling out the survey. The survey consisted of a demographic information section, the Maslach Burnout Inventory, Depression Anxiety Stress Scales (DASS), and a short version of the Copenhagen Psychosocial Questionnaire.

#### Human error assessment and reduction technique

The HEART method was developed by Williams in the United Kingdom in 1985. In this method, it is assumed that human reliability depends on the nature of the performing task (33). The HEART uses a quantitative ergonomic approach to ascertain human reliability, and how likely it is that a process will fail because of human error, for a given job (33, 34). This validated technique was designed to be a relatively quick method for experts to assess human reliability and focus on causal factors that have a significant effect on human performance. HEART uses an underlying assumption that human reliability depends on the nature of the task that the person performs (35). The steps of the HEART are described in Table 1 (33–35). Table 2 presents general tasks and generic error probability of the HEART technique and Table 3 represents a sample of error-producing conditions and their coefficients of the HEART technique.

**Table 1:** The steps of the HEART.

Parameter	Step description
Generic task type (GTT)	Classify each task in terms of its generic human unreliability into one of the nine generic HEART task types using Table 2.
Generic error probability (GEP)	Determine the GEP based on the selected GTT using Table 3.
Error producing condition (EPC)	For each task, identify relevant error-producing conditions (EPC) which may negatively influence performance, and obtain the corresponding coefficient using Table 3.
Assessed proportion of effect	Estimate the impact of each EPC on the task based on the judgment of the evaluator.
Assessed effect	Calculate this assessed effect value for each EPC based on the following formula: Assessed Proportion of Effect +1 × (1 − EPC coefficient)
Human error probability (HEP)	Calculate the overall probability of human error based on the following formula: GEP × Assessed effect 1× Assessed effect 2, etc.

**Table 2:** General tasks and generic error probability.

Task code	Generic task description	Generic error probability (GEP)
A	Completely unfamiliar, the job is executed at speed without having any realistic idea of the possible outcome	0.55
B	Making an attempt to change a system to a new or original state, without supervision or instructions	0.26
C	A complex activity that requires a high level of knowledge and skill	0.16
D	A simple job that can be done very quickly, or without much attention	0.09
E	Everyday job, very done, quick activity with low skill level	0.02
F	Attempt to change a system to a new or original state according to the instructions, with some supervision	0.003
G	Very familiar, well-designed, repetitive, routine work that is done several times an hour, and at a high standard by a highly motivated, highly trained person with experience, and full awareness of the challenges of failure, and with time to repair any error, but without required resources	0.0004
H	The correct response to system commands, even when the supervision is increased or automated. Accurate interpretation of the task requirements	0.00002
M	Miscellaneous task for use when no description can be found	0.03

**Table 3:** Sample of error producing conditions and their coefficients.

	Error producing conditions	Coefficient
1	Lack of familiarity with a situation that is potentially important	17
2	Lack of enough time to identify and correct errors	11
3	High workload, especially in the case of providing additional information at the same time	6
4	Ambiguity in operational standards and guidelines	5
5	Lack of experience	3
6	Inadequacy between the training provided to the job and the training needs of the job and the duties that the person is involved with.	2
7	Unreliable tool	1.6
8	Ambiguous allocation of duties and responsibilities	1.6
9	Low morale of the workforce	1.2
10	Disruption of the normal sleep cycle	1.1
11	Loss of calm in doing the task	1.06

#### Demographical information questionnaire

The demographic information questionnaire asked questions relating to participant’s age, work department, work experience, sex, and education level.

#### Maslach burnout inventory

The MBI was developed by Maslach and Jackson to measure occupational burnout. The MBI has 22 items that evaluate three dimensions of burnout: emotional exhaustion (9 questions), depersonalization (5 questions), and personal accomplishment in a professional context (8 questions) (36). Maslach and Jackson distinguished them and introduced questions related to each of these dimensions (36). All questions were scored using a 7-point Likert scale from 0 to 6, and subscale scores were calculated. For emotional exhaustion and depersonalization, a higher score shows higher burnout; for personal accomplishment, a low score is indicative of burnout. Maslach et al. reported internal consistency coefficients of 0.9 for emotional exhaustion, 0.79 for depersonalization, and 0.71 for personal accomplishment (37). Good psychometric properties of the Persian version of this questionnaire were confirmed by Moalemi et al. (38). They reported Cronbach alphas of 0.85 for emotional exhaustion, 0.71 for depersonalization, and 0.76 for personal achievement (38).

#### Depression anxiety stress scales

The DASS is a concise evaluative instrument consisting of 21 items designed to assess the severity of symptoms associated with depression, anxiety, and stress. It poses seven inquiries for each of the three psychological conditions. Responses are recorded on a four-point Likert scale, ranging from zero to three. To obtain a score for each subscale, a total of the item responses pertinent to that specific domain is calculated. For each subscale, a higher score indicates a more severe manifestation of the disorder in question (39). Scores under 11 suggest a lower presence of these conditions, while those exceeding 11 indicate a higher prevalence. The psychometric properties, including reliability and validity of the DASS in its Persian translation, have been examined within an Iranian cohort (40); Cronbach’s alpha coefficients for the subscales measuring depression, anxiety, and stress were 0.77, 0.79, and 0.78, respectively (40).

#### Copenhagen psychosocial questionnaire

A Persian version of the COPSOQ was used to evaluate work-related psychosocial risk factors. This questionnaire is one of the most comprehensive standard questionnaires that cover a wide range of psychological factors. In this study, we used a version with 39 questions with 18 dimensions that focused on the aim of this study, while omitting dimensions (e.g., Burnout and stress) that could affect the relationships we were examining. Thus, items in the version used included: quantitative demands (4), work pace (3), emotional demand (3), decision authority (1), skill discretion (2), meaning at work (2), predictability (2), rewards (1), role clarity (3), quality of leadership (3), supervisor support (1), co-worker support (2), job satisfaction (1), job insecurity (3), work–family conflict (3), trust (2), justice and respect (2), and self-rated health (1) (41). The participants answered all questions based on one of two types of a five-point Likert scale (always to never, or to a very large extent, to a very small extent), and scored from 0 to 4. A total score was calculated from the sum of all 39 items, in which high scores represent greater psychosocial risk (41). Other Persian versions of the COPSOQ have reported good validity and reliability of this questionnaire. For example, Aminian et al. study yielded a four-factor model with Cronbach’s alpha coefficients ranging from 0.75 to 0.89 (42).

### Statistical analyses

Statistical analyses were conducted using the SPSS software, version 24, to generate descriptive statistics. Descriptive statistics were computed, and according to published cut-off values, or median values of the variables under investigation in this study (error probabilities, emotional exhaustion, depersonalization, personal accomplishment, psychosocial risk factors, depression, anxiety, and stress), such that participants were allocated to either a “low” or a “high” group, where low was the preferred status for all variables. This classification was performed by the median value in the range of scores of the questionnaires.

To model the relationships among the eight variables being examined and the conditional probability at each node, a Bayesian network was constructed using GeNIe academic software, version 2.3. The expectation–maximization algorithm was applied for parameter estimation within the Bayesian network framework. The expectation–maximization algorithm is recognized as a deterministic method for estimation that achieves results asymptotically, making it well-suited for inferring unknown parameters in instances where data may be incomplete or not fully reported (43). Following the creation of the Bayesian network’s theoretical framework, a conditional probability table was derived using the model with the assistance of the expectation–maximization algorithm. Subsequently, delta p sensitivity analyses were performed to assess the impact of various variables (44, 45). To conduct the sensitivity analysis, the probability of one category within the selected variables was set at 100 percent, and the resultant variations in other variables were documented. The sensitivity analysis encompassed all possible states for individual variables as well as combinations thereof. To validate the model’s accuracy, a 10-fold cross-validation method was used. That is, the dataset was divided into ten; nine of the subsets were used to train the Bayesian network, and the tenth subset served to test the validity of the model (46).

## Results

The mean ± standard deviation of the 252 participant’s age was 36.60 ± 7.81 years. The other demographic characteristics of participants are reported in Table 4. The majority of the participants were in the 30–39 years age group (40.9%), male (98.4%), and educated to associate degree level (38.5%). Table 5 presents the distribution of the studied variables according to classification.

**Table 4:** Demographic characteristics of the participants (*N* = 252).

Variable		Frequency	
		N	%
Age (years)	< 30 years	65	25.8
	30–39 years	103	40.9
	40–49 years	77	30.6
	50 + years	7	2.8
Sex	Male	284	98.4
	Female	4	1.6
Education level	Under diploma	6	2.4
	Diploma	61	24.2
	Associate degree	97	38.5
	Bachelor’s degree	67	26.6
	Master’s degree	21	8.3
Job type	Input operator	47	18.7
	Furnace operator	55	21.8
	Production operator	41	16.3
	Input technician	9	3.6
	Furnace technician	10	4.0
	Production technician	23	9.1
	Supervisor	10	4.0
	Repairman	7	2.8
	Employee	11	4.4
	Advisor	2	0.8
	Warehouse keeper	7	2.8
	Security	9	3.6
	Shift manager	2	0.8
	Accountants	5	2.0
	Turner	2	0.8
	Laboratory expert	5	2.0
	Mechanic	5	2.0
	Assembler	2	0.8

**Table 5:** Distribution of the studied variables.

Variable		Frequency	
		N	%
Error probabilities	Low	125	49.6
	High	127	50.4
Psychosocial risk factors	Low	95	37.7
	High	157	62.3
Depression	Low	89	35.3
	High	163	64.7
Anxiety	Low	83	32.9
	High	169	67.1
Stress	Low	97	38.5
	High	155	61.5
Emotional exhaustion	Low	121	48.0
	High	131	52.0
Depersonalization	Low	126	50.0
	High	126	50.0
Reduced personal accomplishment	Low	94	37.3
	High	158	62.7

Low is the desired state for all variables.

Based on the results, the values of Cronbach alpha of emotional exhaustion, depersonalization, and personal achievement were computed by 0.87, 0.89, and 0.90, respectively. The values of Cronbach alpha of depression, anxiety, and stress were also estimated by 0.88, 0.87, and 0.91, respectively. The value of this coefficient for the psychosocial questionnaire was calculated as 0.89. these values were higher than those reported in the previous studies (38, 40, 42).

Table 5 presents the distribution of the studied variables according to classification. The results showed that the majority of the participants had high error probabilities (50.4%), high psychosocial risk factors (62.3%), high depression (64.7%), high anxiety (67.1%), high stress (61.5%), high emotional exhaustion (52.0%), high depersonalization (50.0%), and high reduced personal accomplishment (62.7%). Table 6 is a conditional probability table for error probabilities. Based on the results of the whole conditional probability analyses, participants have properly been distributed in various groups.

**Table 6:** The conditional probability table for burnout on human error probabilities.

Emotional exhaustion	Depersonalization	Reduced personal accomplishment	Error probabilities	
			Low	High
Low	Low	Low	0.906	0.094
		High	0.659	0.341
	High	Low	1.000	0.000
		High	0.500	0.500
High	Low	Low	0.857	0.143
		High	0.363	0.636
	High	Low	0.750	0.250
		High	0.062	0.938

Low is the desired state for all variables.

**Figure 1:**
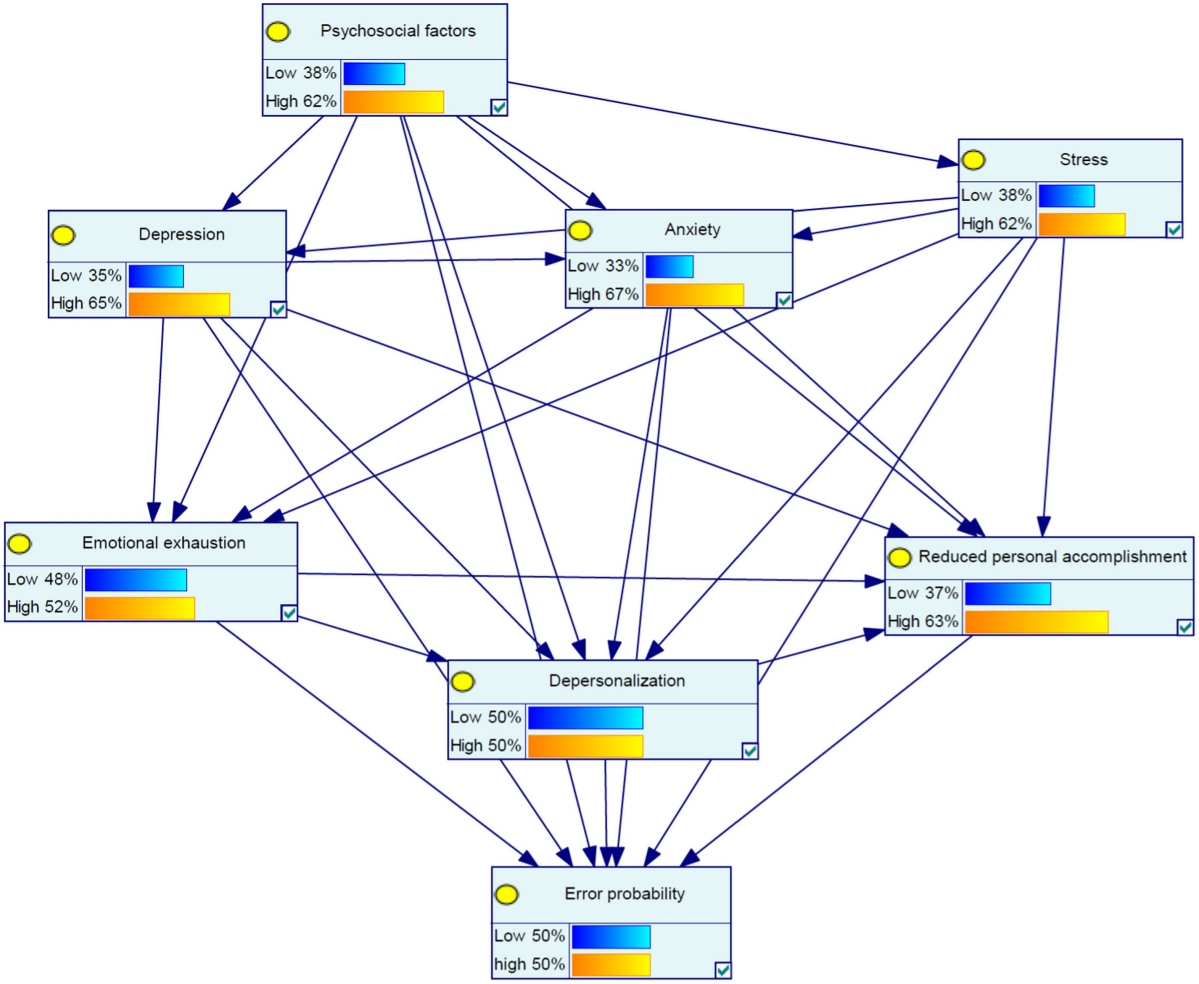
The theoretical model for the marginal probabilities of the seven variables on human error probability according to the Bayesian network model.

Figure 1 shows the theoretical model for the marginal probabilities of the variables according to the Bayesian network model. In this model, the relationships between the studied variables, including psychosocial factors, depression, anxiety, stress, emotional exhaustion, depersonalization, and reduced personal accomplishment were drawn. All possible relationships between these variables were drawn in this figure. Table 7 provides the results of the univariate sensitivity analysis. Based on the results of this analysis, at the high state with a probability of 100% for each of the psychosocial risk, depression, anxiety, stress, emotional exhaustion, depersonalization, and reduced personal accomplishment variables, the probability of high error probabilities positively changed by 14, 15, 16, 17, 29, 28, and 23%, respectively. High error probabilities, with a probability of 100%, also were seen to increase the probability of high psychosocial risk, depression, anxiety, stress, emotional exhaustion, depersonalization, and reduced personal accomplishment variables positively by 17, 18, 20, 20, 29, 27, and 28%, respectively. The highest changes in the burnout dimensions for psychosocial risk, with a probability of 100%, were related to depersonalization (+20), and for the variables depression, anxiety, and stress, with a probability of 100%, were related to emotional exhaustion (+25, +24, and + 30).

**Table 7:** The results of the univariate sensitivity analysis.

Parameter	Level	Low (100%)								High (100%)							
		Psychosocial risk factors	Depression	Anxiety	Stress	Emotional exhaustion	Depersonalization	Reduced personal accomplishment	Error probabilities	Psychosocial risk factors	Depression	Anxiety	Stress	Emotional exhaustion	Depersonalization	Reduced personal accomplishment	Error probabilities
Psychosocial risk factors	Low	-	+34	+29	+31	+22	+25	+15	+16	-	-19	−15	−20	−20	−25	−10	−17
	High	-	−34	−29	−31	−22	−25	−15	−16	-	+19	+15	+20	+20	+25	+10	+17
Depression	Low	+32	-	+59	+52	+34	+31	+19	+19	−19	-	−28	−32	−31	−30	−11	−18
	High	−32	-	−59	−52	−34	−31	−19	−19	+19	-	+28	+32	+31	+30	+11	+18
Anxiety	Low	+26	+55	-	+53	+33	+30	+18	+21	−16	−30	-	−33	−31	−30	−11	−20
	High	−26	−55	-	−53	−33	−30	−18	−21	+16	+30	-	+33	+31	+30	+11	+20
Stress	Low	+33	+56	+62	-	+39	+35	+20	+21	−19	−30	−30	-	−35	−34	−11	−20
	High	−33	−56	−62	-	−39	−35	−20	−21	+19	+30	+30	-	+35	+34	+11	+20
Emotional exhaustion	Low	+28	+46	+48	+48	-	+38	+28	+30	−17	−25	−24	−30	-	+38	−16	−29
	High	−28	−46	−48	−48	-	−38	−28	−30	+17	+25	+24	+30	-	+38	+16	+29
Depersonalization	Low	+33	+43	+45	+45	+39	-	+26	+28	−20	−24	−22	−28	−36	-	−15	−27
	High	−33	−43	−45	−45	−39	-	−26	−28	+20	+24	+22	+28	+36	-	+15	+27
Reduced personal accomplishment	Low	+16	+20	+21	+20	+22	+19	-	+29	−9	−11	−10	−12	−19	−19	-	−28
	High	−16	−20	−21	−20	−22	−19	-	−29	+9	+11	+10	+12	+19	+19	-	+28
Error probabilities	Low	+22	+26	+31	+26	+30	+27	+38	-	−14	−15	−16	−17	−29	−28	−23	-
	High	−22	−26	−31	−26	−30	−27	−38	-	+14	+15	+16	+17	+29	+28	+23	-

Low is the desired state for all variables.

Table 8 reports the results of the multivariate sensitivity analysis. At the two variables level, with a probability of 100%, the highest increases in high error probabilities were related to emotional exhaustion and reduced personal accomplishment (41%), and depersonalization and reduced personal accomplishment (41%). At the three variables level, with a probability of 100%, the highest increase in high error probabilities belonged to high psychosocial risk, emotional exhaustion, and reduced personal accomplishment (46%). At the four variables level, with a probability of 100%, the highest increase in high error probabilities belonged to high psychosocial risk, emotional exhaustion, depersonalization, and reduced personal accomplishment (46%). And all variables, with a probability of 100%, increased the probability of high error probabilities by 46%.

**Table 8:** The results of the multivariate sensitivity analyses.

Distribution (100%)							Error probabilities (%)	
Psychosocial risk factors	Depression	Anxiety	Stress	Emotional exhaustion	Depersonalization	Reduced personal accomplishment	Low	High
✓	✓						−19	+19
✓		✓					−21	+21
✓			✓				−22	+22
✓				✓			−33	+33
✓					✓		−30	+30
✓						✓	−33	+33
	✓	✓					−16	+16
	✓		✓				−18	+18
	✓			✓			−29	+29
	✓				✓		−28	+28
	✓					✓	−34	+34
		✓	✓				−17	+17
		✓		✓			−29	+29
		✓			✓		−29	+29
		✓				✓	−35	+35
			✓	✓			−30	+30
			✓		✓		−28	+28
			✓			✓	−36	+36
				✓	✓		−34	+34
				✓		✓	−41	+41
					✓	✓	−41	+41
✓	✓	✓					−20	+20
✓	✓		✓				−21	+21
✓	✓			✓			−33	+33
✓	✓				✓		−30	+30
✓	✓					✓	−37	+37
✓		✓	✓				−22	+22
✓		✓		✓			−33	+33
✓		✓			✓		−31	+31
✓		✓				✓	−39	+39
✓			✓	✓			−33	+33
✓			✓		✓		−31	+31
✓			✓			✓	−40	+40
✓				✓	✓		−37	+37
✓				✓		✓	−46	+46
✓					✓	✓	−44	+44
	✓	✓	✓				−18	+18
	✓	✓		✓			−29	+29
	✓	✓			✓		−28	+28
	✓	✓				✓	−35	+35
	✓		✓	✓			−29	+29
	✓		✓		✓		−28	+28
	✓		✓			✓	−37	+37
	✓			✓	✓		−34	+34
	✓			✓		✓	−40	+40
	✓				✓	✓	−43	+43
		✓	✓	✓			−30	+30
		✓	✓		✓		−28	+28
		✓	✓			✓	−36	+36
		✓		✓	✓		−34	+34
		✓		✓		✓	−41	+41
		✓			✓	✓	−43	+43
			✓	✓	✓		−34	+34
			✓	✓		✓	−41	+41
			✓		✓	✓	−43	+43
				✓	✓	✓	−44	+44
✓	✓	✓	✓				−21	+21
✓	✓	✓		✓			−23	+23
✓	✓	✓			✓		−30	+30
✓	✓	✓				✓	−39	+39
✓		✓	✓	✓			−33	+33
✓		✓	✓		✓		−31	+31
✓		✓	✓			✓	−40	+40
✓			✓	✓	✓		−37	+37
✓			✓	✓		✓	−45	+45
✓				✓	✓	✓	−46	+46
	✓	✓	✓	✓			−29	+29
	✓	✓	✓		✓		−28	+28
	✓	✓	✓			✓	−37	+37
		✓	✓	✓	✓		−34	+34
		✓	✓	✓		✓	−41	+41
			✓	✓	✓	✓	−44	+44
✓	✓	✓	✓	✓			−33	+33
✓	✓		✓	✓	✓		−37	+37
✓	✓			✓	✓	✓	−46	+46
✓		✓	✓	✓	✓		−37	+37
✓		✓		✓	✓	✓	−46	+46
✓			✓	✓	✓	✓	−46	+46
	✓	✓	✓	✓	✓		−34	+34
	✓	✓		✓	✓	✓	−44	+44
	✓		✓	✓	✓	✓	−44	+44
		✓	✓	✓	✓	✓	−44	+44
✓	✓	✓	✓	✓	✓		−37	+37
✓		✓	✓	✓	✓	✓	−46	+46
✓	✓		✓	✓	✓	✓	−46	+46
✓	✓	✓		✓	✓	✓	−46	+46
✓	✓	✓	✓		✓	✓	−45	+45
✓	✓	✓	✓	✓		✓	−45	+45
✓	✓	✓	✓	✓	✓	✓	−46	+46

Table 9 describes the influence values related to the relationships between the variables in the model represented in Figure 1. The highest influence value for emotional exhaustion was stress (0.482), the highest influence value for depersonalization was emotional exhaustion (0.299), the most influence value for reduced personal accomplishment was anxiety (0.224), and the highest influence value for error probabilities was also anxiety (0.188).

**Table 9:** The influence values related to the relationship between the factors in the model.

Parent	Child	Average	Maximum	Weighted
Anxiety	Emotional exhaustion	0.291	0.5	0.291
Anxiety	Depersonalization	0.221	0.5	0.221
Anxiety	Reduced personal accomplishment	0.224	0.5	0.224
Anxiety	Error probability	0.188	0.789	0.188
Depersonalization	Reduced personal accomplishment	0.187	0.627	0.187
Depersonalization	Error probability	0.167	1	0.167
Depression	Anxiety	0.291	0.735	0.291
Depression	Emotional exhaustion	0.217	0.8	0.217
Depression	Depersonalization	0.105	0.5	0.105
Depression	Reduced personal accomplishment	0.198	0.778	0.198
Depression	Error probability	0.142	1	0.142
Emotional exhaustion	Depersonalization	0.299	0.65	0.299
Emotional exhaustion	Reduced personal accomplishment	0.200	1	0.200
Emotional exhaustion	Error probability	0.167	1	0.167
Psychosocial risk factors	Stress	0.514	0.514	0.514
Psychosocial risk factors	Depression	0.117	0.143	0.117
Psychosocial risk factors	Anxiety	0.125	0.404	0.125
Psychosocial risk factors	Emotional exhaustion	0.218	1	0.218
Psychosocial risk factors	Depersonalization	0.191	0.5	0.191
Psychosocial risk factors	Reduced personal accomplishment	0.182	1	0.182
Psychosocial risk factors	Error probability	0.140	1	0.140
Reduced personal accomplishment	Error probability	0.161	1	0.161
Stress	Depression	0.777	0.803	0.777
Stress	Anxiety	0.66	1	0.66
Stress	Emotional exhaustion	0.482	0.84	0.482
Stress	Depersonalization	0.288	0.65	0.288
Stress	Reduced personal accomplishment	0.202	0.778	0.202
Stress	Error probability	0.167	1	0.167

**Figure 2:**
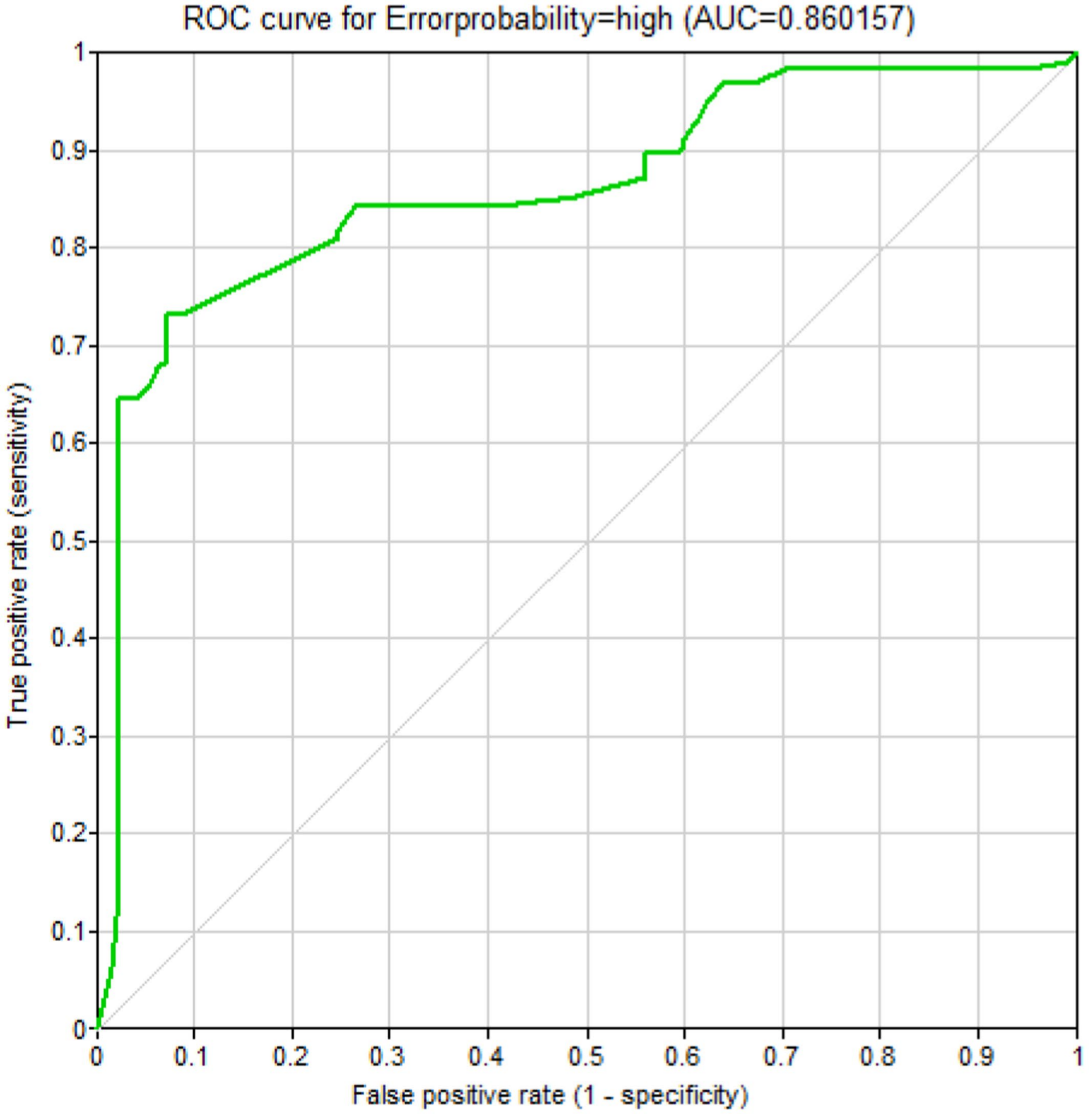
A ROC curve demonstrating the validity of the Bayesian network model.

A receiver operating characteristic (ROC) curve, as a graphical plot, shows the performance of a binary classifier model. A ROC curve drawn to examine the validity of the fitted Bayesian model is shown in Figure 2. Indeed, this curve indicates the ability of the developed model to predict the actual classification. The area under the curve was equal to 0.86. Table 10 presents the confusion matrix related to the classification of the human error status; the values of the sensitivity, specificity, and accuracy of the model were computed as 0.842, 0.736, and 0.821, respectively.

**Table 10:** Confusion matrix related to the classification of the quality of error probability status.

		Predicted		
		Low	High	
Actual	Low	114	11	Sensitivity = 0.842 Specificity = 0.736 Accuracy = 0.821
	High	34	93	

## Discussion

There is extensive literature confirming that human error plays an important role in the occurrence of major accidents (6–9, 47). Thus, the identification of factors that contribute, or in some way influence the occurrence of these errors, is vital to the effective amelioration of this situation. The study reported in this manuscript was conducted for this purpose. The primary aim was to quantify the effect of psychosocial risk factors, mental health factors, and burnout dimensions on human error probabilities in a safety-critical industrial environment, using HEART to quantify task-specific human errors, and a Bayesian networks model to determine probabilities of causal relationships of the eight variables and human error. The results showed that all eight studied variables could significantly affect the distribution of error probabilities. Using a distribution of 100% for the high state of these variables, the results showed that two burnout dimensions made the highest changes in error probability: emotional exhaustion and depersonalization. Regarding mental health, stress was associated with the highest increase in human error probability. Psychosocial risk factors also increased human error probabilities. In the multivariate sensitivity analysis, the highest changes in human error probability in various states were observed with the presence of burnout dimensions, showing the importance of these dimensions to occupational safety.

The results of previous studies had pointed to the importance of burnout on human error. For example, Shanafelt et al. examined the links between burnout dimensions and self-reported medical errors among surgeons and concluded that medical errors have a strong relationship with burnout (48). The results of the present study in the steel industry indicated that high emotional exhaustion and depersonalization could have a stronger causal effect on human error, compared to reduced personal accomplishment—and this was also found in Shanafelt et al.’s study. For a better understanding of these replicated relationships to support intervention, it is helpful to consider how these dimensions contribute to creating burnout in employees. Emotional exhaustion is characterized by a profound sense of being overwhelmed due to the mental exertion associated with one’s professional responsibilities, to the extent that one is unable to interact effectively with people (49). Depersonalization represents another facet of burnout in which individuals experience a sense of emotional and cognitive disengagement from their professional roles and a distant, cynical attitude toward them (49). Reduced personal achievement is reflected in a negative professional self-evaluation and doubts about the ability to perform the job effectively, as well as a greater tendency to evaluate results negatively (50). Given these definitions, emotional exhaustion and depersonalization play a triggering role in the production of burnout. Therefore, these two dimensions can probably influence mental and cognitive performance more strongly than the third dimension, which, in turn, can lead to a higher potential for human errors. This explanation is corroborated by other studies that have specifically mentioned emotional exhaustion and depersonalization as key components of burnout (51). Also, Pehlivanoğlu et al. investigated the effect of emotional exhaustion and depersonalization on personal accomplishment and concluded that there are negative and significant relationships between emotional exhaustion and depersonalization with personal accomplishment (52). Baier et al. investigated the link between worker burnout and safety performance in an emergency medical setting and similarly found that within the spectrum of burnout symptoms, depersonalization, and emotional exhaustion could affect safety behaviors with coefficients of 1.20 and 0.89 (53). Finally, Salyers et al. undertook a meta-analysis of 40 independent samples that explored the connection between burnout and safety within the healthcare sector (54). Although they could not distinguish human error from adverse events, they did find a significant association for all three burnout dimensions and stated that: “The relationship between burnout and safety risk is of particular concern” because the findings are associated with substantial negative outcomes for patients. Our findings strongly suggest, for the first time, that burnout can also have substantial negative consequences in the high-risk safety-critical industrial sector. Interventions to improve occupational safety should include measures to manage the potentials for burnout.

Our study also confirmed that psychological stress could pose a greater risk for human errors compared to other mental health problems. In addition, the Bayesian network model revealed that burnout dimensions could mediate the effect amounts of psychosocial risk factors and mental health problems on human errors. Wang et al. found that stress can disrupt safety participation in workers and that this relationship was mediated by psychological capital (55), and López-Núñez et al. showed that psychological capital has a significant protective factor for burnout (56). Together these studies suggest that effective interventions that serve to improve employees’ resilience and capabilities ultimately ameliorate the potential for work-related stress and burnout, reduce human errors, and improve occupational safety.

The fact that the literature includes studies that confirm that there are correlations between the different variables we were investigating in the context of humans, provided the impetus for this study, which looked out for mediation effects that potentiate human error outcomes using Bayesian networks. This methodology can cope with these relationships to direct causal interpretations. For example, Mousavi et al. reported a significant positive correlation between the burnout dimensions and depression, anxiety, and stress in nurses—replicated in this study (57). In addition to those results, the present study identified anxiety as an important factor affecting error probability in humans. The results of a study performed by Allsop and Gray on the effect of anxiety on attention and behavior indicated that anxiety can negatively influence attentional control (58). Also, Weems et al. concluded that anxiety disorders in young people can lead to cognitive errors (59). Moreover, the multivariate sensitivity analysis in the present study showed that combinations of variables had higher effects on error probability. It is clear that combinations of variables can more strongly impress on a consequence (than looking at bi-variate analyses).

A strength of this study was providing a new context for examining psychosocial factors implicated in causing human error. Most previous investigations of the contribution of burnout, stress, and other mental health and psychosocial risk factors have been undertaken in medical and other healthcare settings, and this study was conducted in the high-hazard, safety-critical steel industry, which is a significant contribution to the literature. We acknowledge some limitations as well. One of the limitations of the present study was that most of the participants were males, and we cannot be sure that these effects are the same between males and females. Meanwhile, the results of a study performed by Prasetyo et al. show that females experience higher job stress than males (60). Also, there are some differences in mental health between females and males. So that females are more prone to mental health disorders in workplaces (61, 62). Liu et al. also concluded that female healthcare workers are more vulnerable to mental health problems (63). These differences need investigation in future research. Moreover, because of the limited number of participants, the effect of sub-scales in the COPSPQ used to assess psychosocial risk factors could not be investigated in the model. It is suggested that these agents are examined in a future study.

## Conclusion

The results of this study showed that all the studied variables, including psychosocial risk factors, mental health problems, and burnout dimensions, can significantly affect the distribution of error probabilities. The most important variables in terms of the effect on error probabilities were the burnout dimensions. Also, the model revealed that burnout dimensions could mediate the size of the effect of psychosocial risk factors and mental health problems on human errors. Therefore, these findings provide direction in terms of the preventive measures needed for controlling human error. These must focus on reducing the prevalence of burnout in workers, which in turn will reduce the effect of psychosocial risk factors and mental health problems on unsafe behaviors.

## Data Availability

The raw data supporting the conclusions of this article will be made available by the authors, without undue reservation.
